# Pricing and reimbursement of orphan drugs: the need for more transparency

**DOI:** 10.1186/1750-1172-6-42

**Published:** 2011-06-17

**Authors:** Steven Simoens

**Affiliations:** 1 Research Centre for Pharmaceutical Care and Pharmaco-economics, Katholieke Universiteit Leuven, O&N2 bus 521, Herestraat 49, 3000 Leuven, Belgium

## Abstract

Pricing and reimbursement of orphan drugs are an issue of high priority for policy makers, legislators, health care professionals, industry leaders, academics and patients. This study aims to conduct a literature review to provide insight into the drivers of orphan drug pricing and reimbursement.

Although orphan drug pricing follows the same economic logic as drug pricing in general, the monopolistic power of orphan drugs results in high prices: a) orphan drugs benefit from a period of marketing exclusivity; b) few alternative health technologies are available; c) third-party payers and patients have limited negotiating power; d) manufacturers attempt to maximise orphan drug prices within the constraints of domestic pricing and reimbursement policies; and e) substantial R&D costs need to be recouped from a small number of patients.

Although these conditions apply to some orphan drugs, they do not apply to all orphan drugs. Indeed, the small number of patients treated with an orphan drug and the limited economic viability of orphan drugs can be questioned in a number of cases. Additionally, manufacturers have an incentive to game the system by artificially creating monopolistic market conditions.

Given their high price for an often modest effectiveness, orphan drugs are unlikely to provide value for money. However, additional criteria are used to inform reimbursement decisions in some countries. These criteria may include: the seriousness of the disease; the availability of other therapies to treat the disease; and the cost to the patient if the medicine is not reimbursed. Therefore, the maximum cost per unit of outcome that a health care payer is willing to pay for a drug could be set higher for orphan drugs to which society attaches a high social value.

There is a need for a transparent and evidence-based approach towards orphan drug pricing and reimbursement. Such an approach should be targeted at demonstrating the relative effectiveness, cost-effectiveness and economic viability of orphan drugs with a view to informing pricing and reimbursement decisions.

## Background

A rare disease is a disease with a very low prevalence. In the European Union (EU), rare diseases are defined as life-threatening or chronically debilitating diseases with a prevalence of 5 out of 10,000 individuals or less [1]. The EU defines an orphan drug as either a medicinal product intended for a life-threatening or chronically debilitating rare disease or a medicinal product that would not be developed without incentives because its sales are unlikely to generate sufficient return on investment. An additional requirement to qualify as an orphan drug is that no satisfactory method exists to diagnose, prevent or treat the disease or, if such a method exists, that the medicinal product will be of significant benefit to those affected by that disease [1].

The EU implemented specific policies in 2000 to stimulate innovation in the field of orphan drugs [1]. Manufacturers that have an orphan designation (i.e. the award of orphan status to a drug) for a medicinal product benefit from: a) protocol assistance (scientific advice during the product development phase); b) direct access to the European Drugs Agency (EMA) Centralised Procedure with respect to registration; c) ten-year marketing exclusivity starting from the date of marketing authorization (i.e. the approval to market a drug); and d) financial incentives (fee reduction or exemptions, possible assistance with research and development). Orphan drug policies can be considered a success as the number of orphan designations and marketing authorizations granted by EMA increased from 270 designations and 22 authorizations by the end of 2005 to 805 designations and 61 authorizations by April 2011 [2].

Whereas decisions surrounding orphan designation and marketing authorization of orphan drugs are taken at the EU level, decisions governing pricing and reimbursement of orphan drugs are a member state responsibility. As a result, evidence requirements, pricing and reimbursement policies governing orphan drugs differ between countries; thereby creating differences in access to, prices and utilization of orphan drugs between countries [3].

Although a detailed discussion of differences between drugs for rare diseases and for common diseases falls outside the scope of this article, the reader should note that the features of rare diseases and orphan drugs make them an issue of high priority for policy makers, researchers, legislators, health care professionals, industry leaders, patients and interest groups. For instance, the present EU system of orphan designation allows for drugs for non-orphan diseases to be designated as orphan drugs. The economic factors underlying orphan designation can be questioned in some cases as a low prevalence of a certain indication does not equal a low return on investment for the drug across its indications. High-quality evidence about clinical added value of orphan drugs is rarely available at the time of marketing authorization due to the low number of patients. Moreover, economic evaluations tend to find that orphan drugs are not cost-effective because the incremental cost for the additional health benefit provided by the orphan drug is usually high. Finally, given the high price of orphan drugs, policy makers are faced with an increasing proportion of pharmaceutical expenditure being spent on orphan drugs [4]. The aim of this study is to conduct a review of the international scientific literature to provide insight into two policy aspects surrounding rare diseases and orphan drugs, i.e. the pricing and reimbursement of orphan drugs. In contrast with a recent study from the industry perspective [5], this review addresses pricing and reimbursement of orphan drugs mainly from the health care payer perspective.

## Pricing

Pricing of orphan drugs follows the same economic logic as drug pricing in general: the price of an orphan drug is set by a manufacturer in an effort to recoup research and development (R&D) costs and to attain a certain profit margin. Additionally, the price takes into account the value of the product to the patient, market conditions (e.g. the existence of alternative health technologies) and the regulatory pricing and reimbursement environment in a country. However, the market for orphan drugs has inherent market failures, thus resulting in high prices due to a number of reasons.

### Monopoly

In the EU, orphan drugs benefit from a period of marketing exclusivity following marketing authorization. Marketing exclusivity gives a monopoly to the manufacturer as no other company is allowed to market the orphan drug during the exclusivity period. The monopolistic power is strengthened by the fact that no alternative health technology exists for many orphan drugs. Additionally, marketing can further boost market power. Under these conditions, manufacturers have an incentive to charge the maximum price for an orphan drug that the market is able to bear. Health care payers have limited negotiating power, often lack information about the cost structure of orphan drugs, and are under pressure from patient advocacy groups and media to accommodate new orphan drugs [6]. As a result, health care payers are often forced to accept the price offered by the manufacturer.

For instance, a recent study compared prices of 28 designated orphan drugs with prices of 16 comparable non-designated drugs for rare disease indications [7]. Price data were based on official hospital prices (per defined daily dose) in Belgium in 2010. Orphan-designated drugs had a higher median price (€ 138.56 - IQR € 483.06) than non-designated drugs (€ 16.55 - IQR € 28.67) for rare disease indications (p < 0.01). The authors concluded that awarding orphan designation status in itself is associated with higher prices for drugs for rare disease indications.

Manufacturers can attempt to create a monopoly market by splitting up a disease into several sub-diseases that qualify as rare diseases (a practice called 'disease sub-setting', 'salami-slicing' or 'disease stratification') [8]. In other words, artificial sub-sets of a common disease are created with a view to qualifying as several rare diseases. The domains of for example pharmacogenomics and oncology are prime targets for creating new rare diseases [9]. Disease stratification may have many benefits for a manufacturer: the company can benefit from measures to stimulate the development of its products, the company creates a monopolistic market where chronically ill patients receive long-term treatment with its orphan drug, the company incurs lower marketing costs as it needs to reach fewer medical specialists, marketing exclusivity erases the possibility of 'me-too' competitors, and the small market reduces the economic viability for generic drugs [10].

The impact of monopolistic market power on prices can also be witnessed in the observation of price increases when a drug with a common indication receives a second, orphan indication. This can be illustrated with the case of sildenafil in Belgium: the orphan drug Revatio^® ^for pulmonary arterial hypertension was more than six times more expensive than Viagra^® ^for erectile dysfunction in 2011.

In the EU, multiple orphan drugs can be authorised to diagnose, prevent or treat a specific rare disease. As a result, the monopolistic power of an orphan drug is sometimes offset by the availability of other products and competitive pressures may reduce prices. For instance, advanced renal cell carcinoma and cystic fibrosis each have ten drugs with designated orphan status [11]. Using available data on the annual cost per patient of reimbursed orphan drugs in Belgium and the availability of alternative health technologies [4], 13 orphan drugs with an alternative had a lower annual cost per patient than 9 orphan drugs without an alternative, although this finding was not statistically significant (independent samples t-test; p = 0.183).

### Price variation between countries

A study compared prices of ten orphan drugs between 25 EU countries [3]. Price data originated from pharmaceutical industry, health authorities, retail or hospital pharmacies, and national databases. The authors noted that some domestic pricing and reimbursement policies provide incentives to maximise prices of orphan drugs. Countries that adhere to free market pharmaceutical pricing generally have higher drug prices and, thus, higher prices for orphan drugs (e.g. Germany) than countries that regulate prices (e.g. Portugal, Spain). Prices of orphan drugs distributed through the hospital pharmacy are not regulated in most European countries, but are negotiated between the manufacturer and an individual hospital. To have a stronger negotiating position, some hospitals jointly purchase orphan drugs from manufacturers. Also, countries such as Belgium, Greece and Italy have imposed price controls on orphan drugs distributed through the hospital pharmacy. Manufacturers are free to set prices of orphan drugs in the United Kingdom, although the Pharmaceutical Price Regulation Scheme (which regulates industry profitability rather than drug prices) [12] and pharmaco-economic guidelines exert downward pressure on prices of orphan drugs. Although the National Institute for Health and Clinical Excellence in England does not frequently appraise orphan drugs, the Scottish Medicines Consortium and the All Wales Medicines Strategy Group do appraise orphan drugs, with the latter for example having specific guidelines for appraising orphan drugs and ultra-orphan drugs [13]. In France, the system of *authorization for temporary use *allows manufacturers to freely set and maximise the price for an orphan drug which will be fully reimbursed.

### Costs of R&D and market access

**Figure 1:**
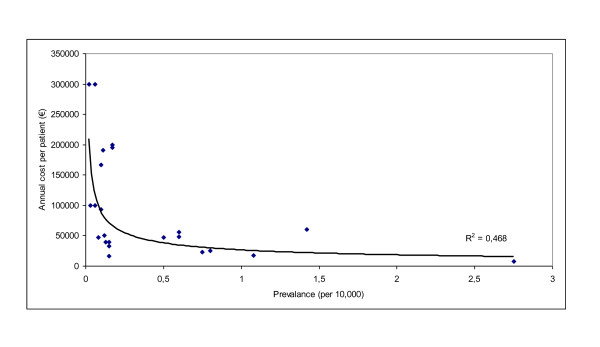
**Association between annual Belgian cost per patient of an orphan drug and disease prevalence**.

The high price of orphan drugs also derives from the cost of the R&D process and of market access procedures. R&D of drugs is a very expensive process associated with a high attrition rate of potential products. Furthermore, in the case of orphan drugs, these R&D costs need to be recouped from a small number of patients, thus resulting in high acquisition costs per patient [14]. A European analysis of orphan drug prices in 25 countries found that the price of an orphan drug is higher for a disease with a lower prevalence [3]. Using published data on the annual cost per patient of an orphan drug in Belgium [4] and the prevalence of the rare disease as derived from Orphanet [15], Figure 1 points to a negative association between the cost of an orphan drug and the prevalence of the disease (y = 26230x^-0,5316^; R^2 ^= 0.468). A similar inverse association has also been observed using Italian data [16].

However, the need to recoup substantial R&D costs from a small number of patients does not apply to all orphan drugs. Some orphan drugs were approved on the basis of historical use, where the manufacturer was not required to produce new evidence on efficacy to gain marketing authorization. In such cases, R&D costs are small. The provision to license drugs without much industry-investment in clinical trials can be illustrated with the case of amifampridine (3,4-diaminopyridine phosphate), which is approved for Lambert-Eaton syndrome. The clinical evidence underpinning this orphan drug primarily referred to the literature on the free base form of 3,4-diaminopyridine [17].

With respect to market access procedures, a recent study showed that orphan drugs were less likely to gain marketing authorization from EMA than other drugs [18]. Furthermore, manufacturers have to comply with different pricing and reimbursement procedures in each member state, thereby raising the price of orphan drugs [10]. Another driver of orphan drug prices occurs following marketing authorization. As many orphan drugs are fast-tracked to market authorization (due to, for example, the life-threatening nature of the disease and the absence of alternative health technologies), regulatory authorities tend to impose expensive post-marketing surveillance programmes.

Many orphan drugs target few patients as they are used to treat rare or ultra-rare diseases, the prevalence of which is generally ill-researched. Also, not all patients are diagnosed or need treatment [5]. Nevertheless, the assertion that orphan drugs target few patients does not apply to all cases [19]. First, certain orphan drugs have proved to be effective against multiple rare diseases and, thus, target a larger number of patients. Examples are sorafenib, which has been approved by the EMA to treat patients with hepatocellular carcinoma and advanced renal cell carcinoma; and imatinib, which has six designated orphan indications in the EU. Second, the use of an orphan drug for a rare disease may subsequently be extended to a common disease. Bosentan, an orphan drug for the treatment of pulmonary arterial hypertension, may also be effective to treat heart failure [20]. Third, a drug for a common disease may subsequently develop an indication for a rare disease. An example is sildenafil, whose initial indication of erectile dysfunction was later extended to include pulmonary artery hypertension and chronic thromboembolic pulmonary hypertension. Fourth, while a disease may qualify as a rare disease in one country, it may be more common in other countries (e.g. drugs to treat tropical diseases). Furthermore, a common disease in one country (e.g. Balkan nephropathy) may qualify as a rare disease at EU level [11]. Finally, although these diseases are individually rare, rare diseases collectively affect approximately 30 million Europeans [6].

### Economic viability

Legislation in the EU attempts to address the issue of the price and the economic viability of orphan drugs. EU legislation is in place to reduce the period of marketing exclusivity if an orphan drug turns out to be sufficiently profitable [1]. However, it is not clear what is meant by 'sufficiently profitable' and this legislation has never been put into practice. The lack of economic viability of orphan drugs can be questioned in certain cases. Some orphan drugs probably do not require a high level of investment to market the drug. For instance, the costs of extending the indication of sildenafil to pulmonary artery hypertension and chronic thromboembolic pulmonary hypertension are likely to be limited to conducting clinical trials and to marketing.

### Orphan biopharmaceuticals

Many orphan drugs (in development) are made by or derived from living organisms using biotechnology. A study focused on ex-manufacturer prices of biopharmaceuticals in five European countries (France, Germany, Italy, Spain, and the United Kingdom), Australia, Canada, Japan, Mexico, and the United States [21]. The authors argued that biopharmaceutical prices may be less regulated and higher than those of chemically-derived drugs given that: a) some countries exclude biopharmaceuticals used in hospital from price regulation; b) price comparisons with other products in a therapeutic class are less likely to occur for biopharmaceuticals with a novel mechanism of action or indication; c) informal cost-effectiveness thresholds may be higher for biopharmaceuticals that address unmet clinical needs or that treat rare diseases; and d) some countries have in place industrial policies to support the development of biopharmaceuticals.

When the 20-year patent on a biopharmaceutical expires, less expensive versions of the drug, so-called biosimilar drugs or follow-on biologics, can enter the market. Orphan biopharmaceuticals tend to face limited competition from biosimilars due to difficulties in and the costs of demonstrating bio-similarity. To substantiate the claim of biosimilarity in Europe, the manufacturer must conduct a direct and extensive comparability exercise between the biosimilar and the reference biopharmaceutical, with a view to demonstrating that the two products have similar quality, safety and efficacy.

## Reimbursement

### Economic evaluation of orphan drugs

Evidence derived from economic evaluations is used to inform pharmaceutical reimbursement (and/or pricing) decisions in many countries. The economic evaluation of orphan drugs is inhibited by the existence of often limited and weak clinical data at launch time. In the context of rare diseases, it may prove difficult to recruit a sufficient number of patients and medical centers in clinical trials, thus raising costs. Orphan drug trials (in for example the field of oncology) may be halted early on ethical grounds when an interim analysis demonstrates clinical superiority of the orphan drug in terms of an intermediate outcome measure such as progression-free survival. It has been recommended to allow greater use of surrogate outcome measures for orphan drugs if clinical data are incomplete, but impose at the same time a commitment to continue research [22].

A central component of this approach is the commitment to ongoing evaluation through, for example, patient registries of rare diseases designed to collect the necessary data to follow up and evaluate uncertainties surrounding the longer-term effectiveness and cost-effectiveness of orphan drugs used in the treatment of rare diseases [23]. Setting up patient and disease registries is part of EU policy and is an action line in rare disease plans that many member states have in place or are setting up. The use of patient registries would support the decision-making process, inform clinical practice, and could provide information about long-term adverse events.

However, patient registries of rare diseases have their limitations. A patient registry may be biased if the patient etiology and disease severity change over time. Also, patient registries tend to collect data on a specific orphan drug used in the treatment of a rare disease, but not on alternative treatments, thus providing partial information to calculate the cost-effectiveness of the orphan drug relative to an alternative treatment. Furthermore, new treatment strategies may become available during the period covered by the registry. Therefore, patient registries of rare diseases need to be set up in a flexible way to collect sufficient data and to account for the evolution in patient population and treatment strategies over their lifecycle.

### Societal considerations

Given their high price for an often modest effectiveness, orphan drugs are unlikely to provide value if their cost-effectiveness ratio is compared to a fixed threshold value (e.g. the threshold of £20,000-£30,000 per QALY used by NICE in England and Wales [24]) [14]. This raises the question of whether society needs to provide incentives to pharmaceutical industry to develop orphan drugs when the costs surpass the value that society attaches to the health benefits produced by orphan drugs [25]? In order to answer this question, it has been argued that other societal considerations may matter when evaluating an orphan drug, such as the observation that orphan drug reimbursement conforms to the principle of social solidarity in which vulnerable groups receive support; that orphan drugs tend to target life-threatening diseases for which there may be no alternative therapy; and that orphan drugs have a considerable impact on patients' health care expenditures if they would have to incur the drug costs themselves. For instance, the Pharmaceutical Benefits Advisory Committee in Australia is reported to take into account such societal considerations [26].

How can these various considerations be aggregated? In other words, how can the often high cost-effectiveness ratio, weak clinical data, small health benefit, high cost and absence of an alternative therapy for orphan drugs be taken into account in a payer's decision to cover such a drug? It has been argued that the threshold ICER should be higher for drugs to which society attaches a high social value [14]. Alternatively, equity weights could be applied to outcome measures according to disease prevalence [27]. Methodological guidance issued by NICE in January 2009 stated that weights should consider the uncertainty surrounding the evidence of the drug's clinical effectiveness and the value that patients place on additional months of life [28]. Weighted outcomes would increase the health gain achieved by an orphan drug, so that there is a higher probability that an orphan drug has an ICER below the threshold value. However, to the best of the author's knowledge, this approach has not been applied in practice yet.

Orphan drugs may attract a high social value, although more research is needed to elicit social values ascribed to orphan drugs and to rare diseases [25]. A literature review has indicated that society attaches a higher value to health improvements experienced by patients who have worse lifetime health prospects [29]. In 2005, NICE in England and Wales set up a Citizen's Council with a view to identifying criteria that the National Health Service may use to value orphan drugs more highly [30]. The top three criteria were the degree of severity of the disease, whether treatment achieved more than just stabilize the disease, and whether the disease was life-threatening. Eighty percent of the Council indicated that disease severity might be a reason to pay a premium for drugs, but disease rarity was not. A Norwegian study explored whether a societal preference existed for giving priority to the treatment of rare diseases and for accepting a higher threshold ICER for orphan drugs [31]. Although respondents supported equal treatment rights for patients with rare diseases, no societal preference for rarity existed if treatment of rare diseases implied that patients with common diseases could not be treated in the context of a finite budget.

The relevance of societal considerations can be illustrated with several orphan drug decisions made by the Medicine Reimbursement Committee in Belgium and NICE in England and Wales. A Belgian study reviewed reimbursement dossiers of 26 orphan drugs submitted between January 2002 and June 2008 [4]. Reimbursement was awarded to 22 orphan drugs. The Medicine Reimbursement Committee advised to reimburse 19 drugs and reimbursement was approved by the Minister of Social Affairs. Although the Committee did not issue an advice relating to one drug, reimbursement was approved by the Minster of Social Affairs. For the remaining two orphan drugs, the dossiers indicated that both the Medicine Reimbursement Committee and the manufacturer proposed a number of elements for negotiation - including a price decrease, employment opportunities, restrictions on the size of the patient population, the funding of diagnostic tests by the company, a reduction of the dosage - which may have played a role in awarding reimbursement. The rationale for not granting reimbursement to four orphan drugs may be related to the high cost of these drugs in comparison with alternative drugs or the existence of other non-orphan indications of the drug.

Despite an unfavorable cost-effectiveness ratio, NICE approved imatinib for the treatment of chronic myeloid leukaemia with an ICER of £37,000 per QALY in the chronic phase, £38,400 per QALY in the accelerated phase and £49,000 per QALY in the blast phase in the absence of any effective alternative therapy (except for bone marrow transplantation) and on equity grounds [32]. A second example relates to enzyme replacement therapy for Fabry's disease. An economic evaluation stated that, although the ICER of enzyme replacement therapy is at least six times higher than the threshold value adopted by NICE, clinicians and manufacturers argued that the National Health Service had no option but to provide this therapy because Fabry's disease is a rare disease [33].

Innovative mechanisms have been proposed for the reimbursement of orphan drugs. Risk-sharing arrangements are schemes in which the manufacturer shares the risk with the health care payer that the product may not be effective for a particular patient. If the product does not have the expected effect, the company may loose some or all product revenue, or needs to provide a replacement product [34]. Such arrangements are instituted at the level of a defined patient population, may require physicians to be trained in the appropriate use of the drug, and necessitate the implementation of a tracking system to follow up its use. For instance, the Scottish Medicines Consortium has in place an Orphan Medicines Risk Share scheme [35]. Medicines included in the risk share scheme are agalsidase alpha and beta for Fabry's disease; imiglucerase and miglustat for type 1 Gaucher's disease; and iloprost for pulmonary arterial hypertension. In England and Wales, NICE has instituted a risk-sharing scheme for the supply of interferon beta and glatiramer acetate which incorporated agreed target treatment effects for patients with multiple sclerosis [36]. If treatment effects were not achieved, the scheme included the option to reduce the drug price to guarantee its cost-effectiveness at a threshold value of £36,000 per QALY. However, some concerns with this scheme have recently been identified [37] and some have argued that the money should be better allocated to funding a randomized controlled trial of interferon beta [27].

## Discussion

In general, the monopolistic power granted to orphan drugs results in high prices. Although these conditions apply to some orphan drugs, it should be emphasised that they do not apply to all orphan drugs. This study has demonstrated that the small number of patients treated with an orphan drug and the limited economic viability of orphan drugs can be questioned in a number of cases. Additionally, manufacturers have an incentive to game the system by artificially creating monopolistic market conditions.

Therefore, there is a need to assess orphan drugs on an individual basis to determine whether their specific features warrant high prices. This assessment should take into account current and planned indications, the existence of alternative health technologies, the total number of patients across registered and off-label indications, and R&D costs. Health care payers could impose the requirement to justify the price based on detailed information about the R&D costs and return on investment at a global level. This could be accompanied by regular monitoring throughout the product's lifecycle. Such an approach necessitates that health care payers make a subjective judgement about an appropriate level of return on investment.

A number of mechanisms to optimise R&D of orphan drugs and to control prices of orphan drugs have been proposed [38]. Auctions of patents have been suggested as a way to reward manufacturers for successfully developing a new orphan drug. Advance purchase commitments entail that the health care payer agrees to pay a specific price for a specified number of units of an orphan drug, thereby guaranteeing a minimum reward for the innovator. Under pay-as-you-go schemes, the health care payer provides additional rewards as a potential drug candidate progresses through the R&D process. Authorities should consider carefully the right incentive strategy for R&D of orphan medicines in rare diseases.

Several European countries have put in place government intervention in rare disease and orphan drug markets with a view to keep down prices, restrict public reimbursement and promote a cost-effective use of orphan drugs [4]. For instance, Belgium, France, Italy and the Netherlands compare the price requested by the manufacturer with the price in other countries. The United Kingdom has set up a system of profit control to constrain prices (although this system will be abolished in favour of a value-based pricing approach from 2013/14) and Sweden uses a system of public procurement at the regional level in order to maximise price competition.

To gain reimbursement, a formal economic evaluation needs to be performed in some, but not all European countries. It should be noted that national authorities tend to demand data on the effectiveness of orphan drugs in a real-world setting rather than on their efficacy in a structured setting. Also, as the cost-effectiveness of an orphan drug is calculated relative to a relevant comparator, there is a need for comparative data. Both factors have implications for the design of patient and disease registries. For orphan drugs that provide a first-in-class therapy for unmet clinical needs, the cost-effectiveness can be calculated on the basis of studies comparing the orphan drug with placebo. For orphan drugs that are marketed in the presence of competitor health technologies, there is a need to compute the cost-effectiveness based on head-to-head studies of the orphan drug relative to a relevant comparator.

## Conclusions

This study has identified and discussed several factors affecting pricing and reimbursement of orphan drugs. There is a need for a transparent and evidence-based approach towards pricing and reimbursement of orphan drugs. Such an approach should be targeted at demonstrating the relative effectiveness, cost-effectiveness, cost structure and economic viability of orphan drugs with a view to informing pricing and reimbursement decisions.

## Competing interests

The author has no conflicts of interest that are relevant to the content of this manuscript.

## Authors' contributions

SS designed the study, carried out the literature review and drafted the manuscript.
